# Assessment of silent spaces at different PEEP levels by electrical impedance tomography in severe COPD

**DOI:** 10.1186/2197-425X-3-S1-A456

**Published:** 2015-10-01

**Authors:** AD Waldmann, PL Róka, SH Bohm, W Windisch, S Strassmann, C Karagiannidis

**Affiliations:** Swisstom AG, Landquart, Switzerland; Budapest University of Technology and Economics, Budapest, Hungary; Department of Pneumology and Critical Care Medicine, Kliniken der Stadt Köln, Cologne, Germany

## Introduction

Electrical impedance tomography (EIT) is a novel method to monitor regional lung function. For this purpose, 32 surface electrodes are placed around the human thorax. Weak alternating currents are applied via two of these electrodes and the resulting potentials are measured at the remaining electrodes. From the measured voltages, real-time images are calculated which show the distribution of electrical impedance within the body representing functions rather than structures. Using EIT these lungs can be analysed on a regional basis with respect to the following risk factors: collapse, at risk of becoming atelectatic or overdistension. Identifying these lung areas of particular clinical relevance by EIT may help to find the best individual PEEP level for each patient.

## Methods

In five mechanically ventilated COPD patients we performed EIT measurements at PEEP 6, 8, 10, 12, 14, 16 cmH_2_O using Swisstom BB^2^ (Swisstom, Landquart, Switzerland). The electrode belt was placed along the sixed intercostal space and based on the measured voltages, tomographic impedance images were calculated. Patient-specific regions of interest (ROI) were selected according to the patient’s height, weight and gender. Tidal variation of impedance within the ROIs was measured as the difference between the end of inspiration and the beginning of inspiration. Maximal pixel amplitude within the tidal image was divided by 10, which results in 10 different amplitude categories according to which each pixel was then assigned. Thereafter, a virtual line perpendicular to the gravity vector through the geometric focal point of overall ventilation (Centre of Ventilation) was defined for each breath as the “ventilation horizon” (Table 1). All pixels lying below this horizon and belonging to the lowermost category were defined as dependent silent space (DSS). The number of these pixels was counted and expressed as % of all pixels within the ROI. Accordingly, the non-dependent silent space (NSS) values describe the percentage of poorly ventilated areas physically located above the ventilation horizon.

## Results

Dependent silent space decreases from 15+/-8% (Mean+/-SD) at PEEP 6 to 5+/-5% at PEEP 16. At the same time the non-dependent silent space increases slightly from 2+/-2% to 6+/-3%.

## Conclusions

We describe the non-dependent and dependent silent spaces during a PEEP titration in 5 patients. The location of dependent silent spaces illustrates the recruitment of none or poorly ventilated areas with increasing levels of PEEP even in mechanically ventilated patients with severe COPD.

**Figure 1:**
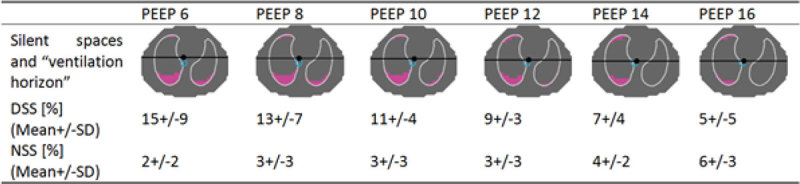
**Dependent and non-dependent silent spaces.**

